# Vortex flow during early and late left ventricular filling in normal subjects: quantitative characterization using retrospectively-gated 4D flow cardiovascular magnetic resonance and three-dimensional vortex core analysis

**DOI:** 10.1186/s12968-014-0078-9

**Published:** 2014-09-27

**Authors:** Mohammed S M Elbaz, Emmeline E Calkoen, Jos J M Westenberg, Boudewijn P F Lelieveldt, Arno A W Roest, Rob J van der Geest

**Affiliations:** Division of Image Processing, Department of Radiology, Leiden University Medical Center, Leiden, C3-Q room 54, Albinusdreef 2, Leiden, 2333 ZA The Netherlands; Department of Paediatric Cardiology, Leiden University Medical Center, Leiden, The Netherlands; Department of Intelligent Systems, Delft University of Technology, Delft, The Netherlands

**Keywords:** Vortex flow, Vortex quantification, 4D Flow, Cardiovascular magnetic resonance, Left Ventricular diastolic function, Intra-cardiac blood flow patterns, Transmitral blood flow

## Abstract

**Background:**

LV diastolic vortex formation has been suggested to critically contribute to efficient blood pumping function, while altered vortex formation has been associated with LV pathologies. Therefore, quantitative characterization of vortex flow might provide a novel objective tool for evaluating LV function. The objectives of this study were 1) assess feasibility of vortex flow analysis during both early and late diastolic filling in vivo in normal subjects using 4D Flow cardiovascular magnetic resonance (CMR) with retrospective cardiac gating and 3D vortex core analysis 2) establish normal quantitative parameters characterizing 3D LV vortex flow during both early and late ventricular filling in normal subjects.

**Methods:**

With full ethical approval, twenty-four healthy volunteers (mean age: 20±10 years) underwent whole-heart 4D Flow CMR. The Lambda2-method was used to extract 3D LV vortex ring cores from the blood flow velocity field during early (E) and late (A) diastolic filling. The 3D location of the center of vortex ring core was characterized using cylindrical cardiac coordinates (Circumferential, Longitudinal (L), Radial (R)). Comparison between E and A filling was done with a paired T-test. The orientation of the vortex ring core was measured and the ring shape was quantified by the circularity index (CI). Finally, the Spearman’s correlation between the shapes of mitral inflow pattern and formed vortex ring cores was tested.

**Results:**

Distinct E- and A-vortex ring cores were observed with centers of A-vortex rings significantly closer to the mitral valve annulus (E-vortex L=0.19±0.04 versus A-vortex L=0.15±0.05; p=0.0001), closer to the ventricle’s long-axis (E-vortex: R=0.27±0.07, A-vortex: R=0.20±0.09, p=0.048) and more elliptical in shape (E-vortex: CI=0.79±0.09, A-vortex: CI=0.57±0.06; <0.001) compared to E-vortex. The circumferential location and orientation relative to LV long-axis for both E- and A-vortex ring cores were similar. Good to strong correlation was found between vortex shape and mitral inflow shape through both the annulus (r=0.66) and leaflet tips (r=0.83).

**Conclusions:**

Quantitative characterization and comparison of 3D vortex rings in LV inflow during both early and late diastolic phases is feasible in normal subjects using retrospectively-gated 4D Flow CMR, with distinct differences between early and late diastolic vortex rings.

**Electronic supplementary material:**

The online version of this article (doi:10.1186/s12968-014-0078-9) contains supplementary material, which is available to authorized users.

## Background

Vortex formation within the left ventricular (LV) blood flow has been suggested to critically contribute to efficient blood pumping function [1]. A vortex can be described as a group of fluid particles with a swirling motion around a common axis. Among different types of vortices, vortex rings (also known as toroidal vortex) are abundant in nature because of their compactness and stability [1-3].

In the LV, in healthy subjects, both *in vivo* and *in vitro* studies have reported vortex ring formation during early diastolic filling, originating at the distal tip of the mitral valve (MV) leaflets [1,4-11]. In a three dimensional (3D) view, this vortex ring appears as a closed tube with torus-like shape distal to the mitral valve orifice. In a two dimensional (2D) four-chamber view a 3D vortex ring appears as a counter-rotating vortex pair, one distal to the anterior MV leaflet and another distal to the posterior leaflet. Such vortex formation may help in efficient MV closure [5], efficient diastolic filling, minimizing kinetic energy loss [4,6] and preventing thrombus formation [7]. An altered (early filling) vortex formation have been shown to develop in patients with diastolic dysfunction and dilated ischemic cardiomyopathy, suggesting a relation between abnormal vortex formation and LV dysfunction [7,8]. On the other hand, in normal subjects, discrepancies arise in literature and little is known about vortex formation during late filling. Experimental studies using computational fluid dynamics (CFD)-based simulations of LV inflow have reported the formation of a vortex ring distal to the MV during late LV filling, [12-17]. In contrast, *in vivo* studies have reported only the formation of a single anterior vortex during late filling (i.e. not a vortex ring because of the absence of a posterior vortex) [6,9,18-21] or even the absence of any vortex [18]. While CFD simulation can provide higher temporal and spatial resolution than *in vivo* data, application of CFD techniques also involve simplifications of the geometry and dynamics of the left ventricle and mitral valve leaflets which might result in inaccurate modelling of the true blood flow.

4D Flow cardiovascular magnetic resonance (CMR) with retrospective cardiac gating can acquire all the three directional velocity components (in-plane and through-plane) of the blood flow relative to the three spatial dimensions and over the whole cardiac cycle, providing a powerful tool for evaluating blood flow patterns during both early and late left ventricular filling *in-vivo* [6,22,23]. Previous studies have shown the feasibility of using 4D Flow CMR for vortex flow analysis [6,19,22,24-27]. These studies mainly focus on vortex formation during early filling inflow but not late filling inflow. While paramount for establishing normal ranges defining LV vortex flow, standardized quantitative characterization of the 3D shape and location of normal vortex flow are currently lacking.

Different from visualization-based vortex identification, vortex core detection techniques [28-30] base their vortex identification on the underlying physical properties of a vortex instead of only visual assessment, therefore, provide more objective vortex definition. CFD experiments have shown that LV vortex ring originates from the inlet jet through the mitral valve orifice during early LV filling [1,4,10,13], therefore, the shape of the formed vortex is expected to resemble the shape of the originating valvular opening [4,13]. Hence, we hypothesized that similar behavior could be identified *in vivo* where a more oval opening of the MV during peak late filling results in a more elliptical vortex ring compared to the one originating from a more circular valve opening during peak early filling. Accordingly, the aims of our study were to apply retrospectively-gated 4D Flow CMR and quantitative 3D vortex core analysis to 1) Assess feasibility of *in vivo* vortex flow analysis during both early and late diastolic filling in normal subjects 2) Establish normative quantitative parameters characterizing 3D LV vortex flow during both early and late ventricular filling in normal subjects.

## Methods

### Study population

Twenty-four healthy volunteers (9 males, mean age 20±10 years; age range 9–44 years), without history of cardiac disease, abnormalities on ECG or echocardiography were included. The study protocol was approved by the institutional review board and written informed consent was given by all subjects or their legal representatives.

### 4D Flow CMR protocol

All subjects underwent 4D Flow CMR using a 3T digital broadband multi-transmit CMR system (Ingenia, Philips Medical Systems, Best, The Netherlands), with maximal gradient amplitude 45 mT/m and maximal slew rate 200 T/m/s. For signal reception, a 60cm Torso coil was used in combination with the FlexCoverage Posterior coil in the tabletop, combining a maximum of 32 elements. A 3D time-resolved volume acquisition of the whole heart was performed with velocity encoding in all three directions with velocity sensitivity (VENC) of 150 cm/s. The acquired volume data was reconstructed in time-resolved manner (30 cardiac phases per cardiac cycle) into 2.3×2.3×3–4.2mm^3^ (three subjects were scanned with 3 mm slice thickness). Retrospective cardiac gating was performed with Vector ECG triggering. Scan parameters: echo time 3.0 ms, repetition time 9.9 ms, flip angle 10°, field-of-view 400 mm, number of signal averages 1. VENC 150 cm/sec. Acceleration was achieved by Echo Planar Imaging with EPI factor 5. Free breathing was allowed and no respiratory motion compensation was used. Commercially available concomitant gradient correction was used for phase offset correction.

### 3D vortex core identification using the Lambda2-method

In this study, vortex cores in the LV cavity were detected over the diastolic phases using the Lambda2 (λ_2_)-method [28]. The Lambda2-method is an objective method that identifies 3D vortex cores based on their physical fluid dynamics properties, and is considered the most accepted vortex detection technique [31]. In short, the Lambda2-method uses the fluid’s velocity gradient properties to obtain a scalar value, λ_2_. In a loose sense, this obtained scalar reflects the pressure due to velocity gradients after excluding the effect of the irrotational part of the flow. The vortex cores are then identified as the regions with extreme negative λ_2_-values. These identified vortex cores can be visualized by use of isosurfaces of isovalue ${\mathrm{T}}_{\lambda_2}$_,_ which is an application-dependent threshold. More technical background of applying the Lambda2-method on 4D Flow CMR, including the choice of the isovalue threshold, has been described earlier [32].

### Vortex core analysis workflow

4D Flow CMR data were analyzed with in-house developed software based on Matlab (Version R2012a, Mathworks Inc., Novi, MI). First, the LV endocardial boundaries were manually delineated using MASS research software (Version 2013EXP, Leiden University Medical Center, Leiden, The Netherlands). Subsequently, the Lambda2-method was applied to the 4D Flow CMR data to identify the vortex structures within the LV blood pool. Early (E) filling and atrial (A) filling phases were defined from the flow rate-time graph after transmitral velocity mapping in combination with retrospective valve tracking [33]. For every subject, the vortex ring core (if detected) of peak early filling and peak late filling was used for further quantitative analysis. As described in previous work, the Lambda2 isovalue threshold (${\mathrm{T}}_{\lambda_2}$) was defined as ${\mathrm{T}}_{\lambda_2}=\mathbf{K}\upmu$ (with **K** as a real number and μ as the λ_2_ average of voxels with λ_2_ < 0) with **K** chosen as the value providing the most circular vortex ring core having the least attached trailing structures [32]. The parameter **K** was chosen separately for every filling phase. The shape and location of the peak early (E) and late (A) -vortex ring cores were further quantitatively analyzed using the parameters explained below. In the remainder, the vortex cores detected at peak early filling and peak late filling will be denoted as E-vortex and A-vortex, respectively.

### 3D quantitative characterization of diastolic vortex ring core

**Figure 1:**
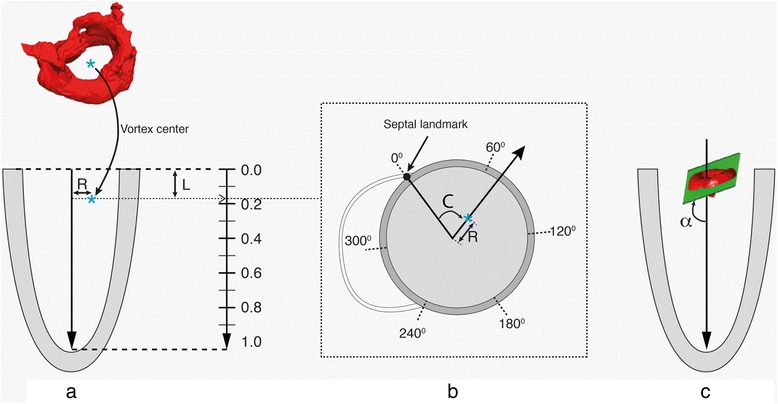
**Definition of the local cardiac coordinate system (C, L, R) relative to the LV: The LV long-axis is defined as the line from the mid of the mitral valvular opening to the LV apex.** The long-axis was calculated separately per filling phase (i.e. one for early filling and another for late filling). The center of the vortex ring was projected on this long-axis. The distance of the projected point to the MV and to the vortex center defined the vortex’s longitudinal (L) and radial (R) coordinates as illustrated in **(a)**, respectively. Both L and R distances were normalized to the long-axis length and to the basal endocardial radius (measured on a reformatted short-axis slice), respectively to provide dimensionless parameters. Circumferential (C) Coordinate is defined as the angle between the septal landmark (the anterior attachment of the RV free wall with the LV) and the vortex center as illustrated in cross-sectional view **(b)**. the vortex ring orientation (α) measured as angle between the LV long-axis vortex and a fitting plane of the vortex ring, where an orientation of 90° means a vortex ring is perpendicular to the LV long-axis as shown in **(c)**.

**Figure 2:**
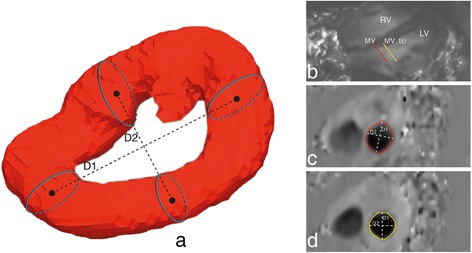
**Diagram showing the measurement of the vortex circularity Index (CI).** In **(a)**, CI=D2/D1, D1 represents vortex’s long diameter and D2 represents the vortex’s shortest diameter. Both diameters measured as the distance between centers of corresponding and opposite cross-sections along the diameter of interest. **(b)** Two planes positioned on through-plane velocity-encoded MR images at the annulus level (red) and one centimeter distal of the annulus (yellow) resulted in two cross-sectional images of the through-plane velocity **(c, d)** in which the flow through MV was outlined and used to define circularity index of MV flow.

The 3D location and orientation of the vortex ring core were quantified using a standardized 3D local cardiac (cylindrical) coordinate system, abbreviated by CLR. Every vortex ring core center was localized using its circumferential (C), longitudinal (L) and radial (R) coordinates and orientation relative to the LV as defined and illustrated in (Figure 1). The shapes of the vortex ring cores were quantified using a dimensionless circularity index (CI) defined as the ratio between the vortex’s short (*D*1) to long (*D*1) diameters, i.e., CI = D1/D2 (See Figure 2a).

### Intra and interobserver reproducibility

One observer repeated the same measurements after one week to allow assessment of intraobserver reproducibility. Two independent observers repeatedly performed measurements on all subjects to assess interobserver reproducibility of derived parameters. The observers manually defined the Lambda2 threshold (${\mathrm{T}}_{\lambda_2}$) as explained above. Then, C,L,R coordinates and orientation of vortex ring cores for both early and late filling were quantified.

### Vortex-Mitral flow association

To investigate the relationship between the geometry of the vortex ring core and the inflow jet area through the MV, the area of MV opening was assessed at two levels using retrospective valve tracking [33]. In short, at the same phase as the selected vortex, a plane was positioned at the annulus level and a second plane at one centimeter distal to the annulus (Figure 2: (b)) as an approximation of the tip level of the opened MV leaflets. These planes resulted in two cross-sectional images with through-plane velocity encoding in which the flow through the opened MV was outlined (Figure 2: (c), (d)). The outlined regions were then used to calculate the circularity index of the inflow area at the mitral annulus level (CI_MV_) and at the valve tip level (CI_MV_tip_), as the ratio between the short- to long-diameters of the outlined region. The correlation between the vortex circularity index *CI*_vortex_ of the diastolic vortex ring cores (E- and A- vortex ring cores pooled together) and each of *CI*_MV_ and *CI*_MV _ tip_ were then evaluated.

### Statistical analysis

Statistical analysis was performed using SPSS Statistics software (version 20.0 IBM SPSS, Chicago, Illinois). Quantitative parameters were presented as mean ± standard deviation or median and inter-quartile ranges (IQR) where appropriate. Differences between E- vortex ring and A-vortex ring parameters were compared using paired Student t-test. Spearman’s correlation test was used to assess the relationship between vortex ring shape and mitral inflow area shape. Inter- and intraobserver reproducibility were determined by the interclass correlation coefficient for absolute agreement, the absolute and relative unsigned difference between measurements (with paired t-test) and the coefficients of variance defined as the standard deviation of the difference divided by the mean of both measurements. A p-value <0.05 was considered statistically significant.

## Results

### Subject characteristics

Clinical characteristics of the study population are shown in Table 1. Three subjects with an absent A-vortex ring core are described separately.

**Table 1:** **Study characteristics**

	**21 subjects**	**Subject A**	**Subject B**	**Subject C**	**Total**
age (years)	21±10	10	9	13	20±10
male/female	8/13	female	male	female	9/15
heart rate (bpm)	69±11	90	107	91	73±14
diastasis duration (ms)	108±73	22	0	0	95±77
E/A ratio	2.6±0.8	2.3	1.83	1.95	2.5±0.7

### Characterization of 3D LV vortex ring cores

**Figure 3:**
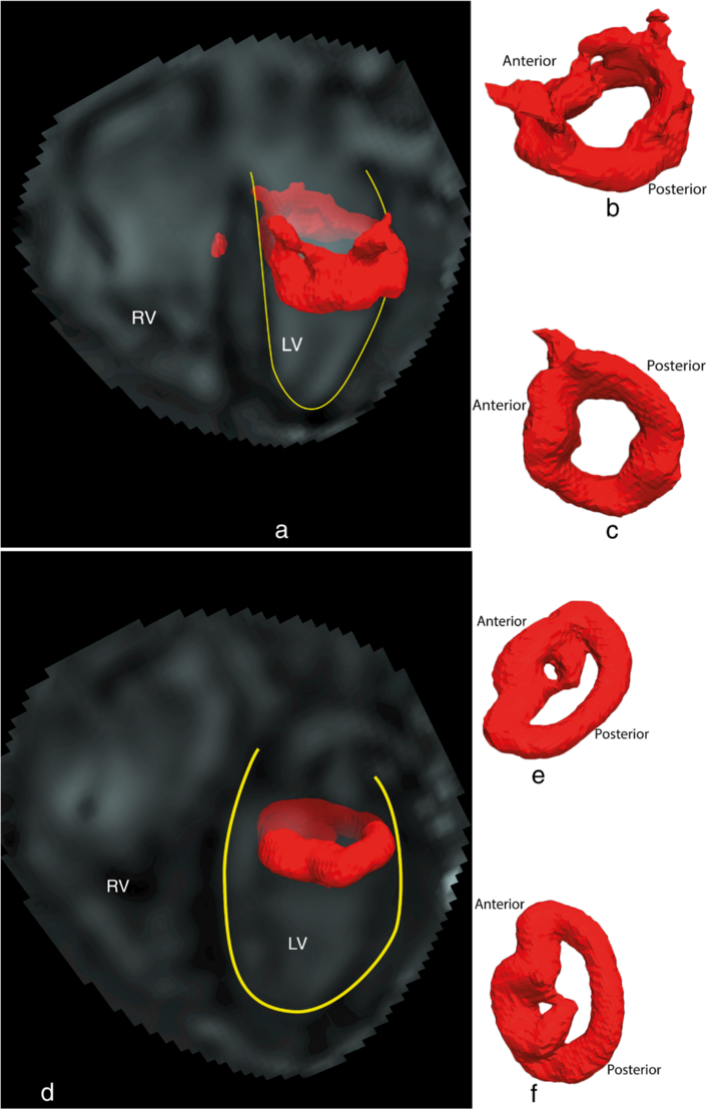
**Results of Lambda2-based vortex core detection from a sample subject:** **(a) Identified vortex ring core at peak early** **(a) diastolic filling with respective location to LV** **(a), in top-down** **(b) and bottom-up (c) views.** Similarly, identified peak late diastolic vortex ring core is shown in **(d)**, **(e)** and **(f)**. The core of the peak early filling vortex ring appears with a quasi-torus-like shape, more circular and symmetrical compared to the core of peak late filling vortex ring which appears more elliptical in shape and asymmetrical with dilated anterior side and compressed posterior side. Lambda2 isovalue threshold $\left({\mathrm{T}}_{\lambda_2}\right)=3\upmu$ was used to define the isosurfaces of vortex ring cores (with μ as the λ_2_ average of voxels with λ_2_ < 0).

**Figure 4:**
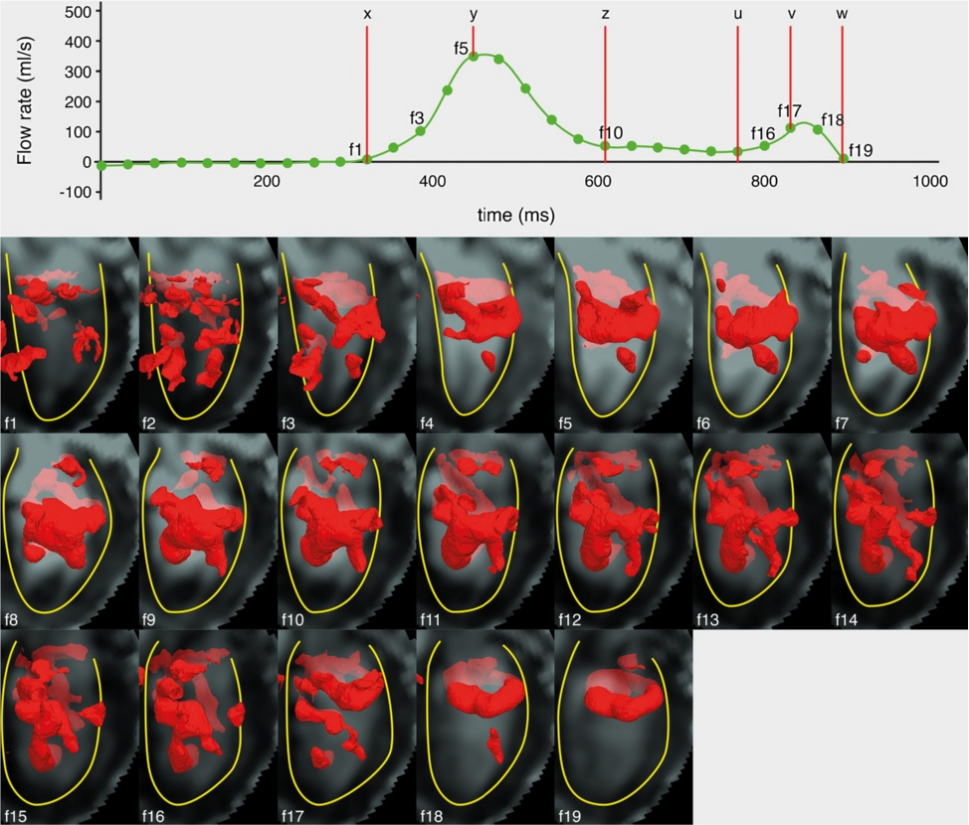
**Time-sequence of the Lambda2-detected 3D LV vortex structures (visualized as isosurfaces in red color) over all acquired diastolic phases of a sample normal subject, with E-filling onset (x), peak (y) and end (z), and A-filling onset (u), peak (v) and end (w).** Diastasis is the duration between z and u. Every dot in the cardiac curve corresponds to a time point of the cardiac cycle in which a 4D Flow volume was acquired. With the start of diastolic phase (f1),the start of the presence of a compact ring-like shaped vortex ring during early- (f3) and late (f7) diastolic filling, the most developed vortex ring formed during early-(f5) and A-filling (f18), the start of vortex stretching or elongation in direction parallel to the LV long-axis (f10) and end of late filling while compact vortex ring is still identifiable (f19). Lambda2 isovalue threshold $\left({\mathrm{T}}_{\lambda_2}\right)=3\upmu$ was used to define the isosurfaces of vortex ring cores (with μ as the λ_2_ average of voxels with λ_2_ < 0). To avoid cluttered view, only large scale vortex cores of 1 cm^3^ or larger are visualized.

**Figure 5:**
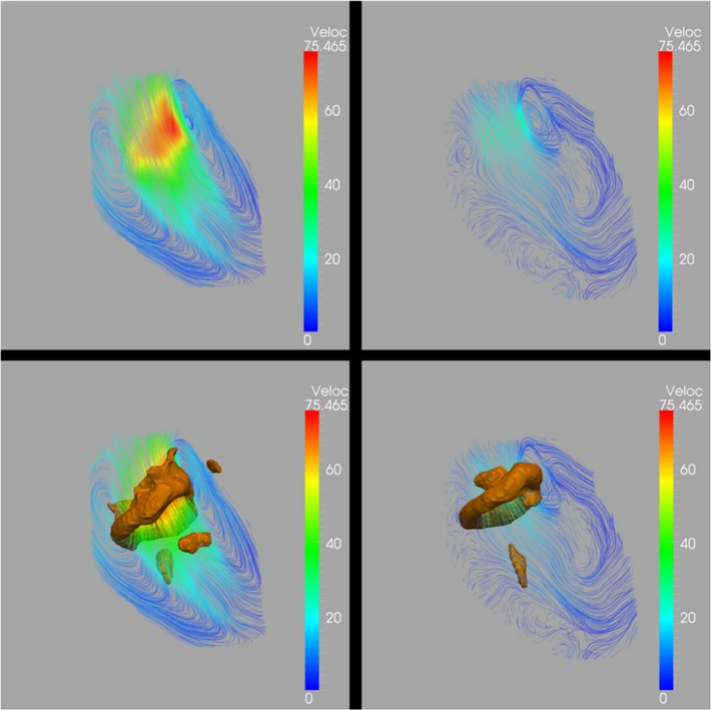
**Streamline superimposed on vortex cores.** Sample Streamline visualization of cross-sectional view of LV flow during peak early filling **(a)** peak late filling **(b)** showing pair of counter-rotating vortices. Streamlines are color coded (blue to red) based on velocity magnitude. Same frames were superimposed with 3D vortex ring cores identified using Lambda2-method and showing good overlap between the 3D Lambda2-detected vortex cores and the cores of corresponding 2D streamlines’ counter-rotating vortices during both peak early **(c)** and peak late filling **(d)**. Lambda2 isovalue threshold $\left({\mathrm{T}}_{\lambda_2}\right)=3\upmu$ was used to define the isosurfaces of vortex ring cores (with μ as the λ_2_ average of voxels with λ_2_ < 0).

In all twenty-four subjects, during the E-filling, a compact quasi-torus-shaped vortex ring core (Figure 3: (a-c)) started to form distal to the mitral valve leaflets shortly after the onset of the E-filling and continued its development during the period of E-filling acceleration, reaching its full development with the E-filling approaching its peak (Figure 4: f1-f5). During E-filling deceleration and diastasis, the vortex core deformed into a complex shape which tended to align with the LV long-axis (Figure 4: f6-f10) while progressing towards the apex. Only a remaining residual of the vortex ring core, located at the mid-ventricular level could be observed at the onset of atrial contraction and this remnant of the E-vortex ring core could not be observed anymore at end diastole (Figure 4: f17,f18). In the majority of subjects (twenty-one subjects, 88%), during the late diastolic filling, a new isolated compact and more asymmetrically shaped vortex ring core was formed at the ventricular basal level with a more dilated anterior side (i.e., the part close to the aortic outflow tract) and more compressed posterior side (Figure 3: (d-f)), reaching its complete formation while approaching peak late filling (Figure 4: f15-f17). The A-vortex ring core was persistently present until the end of diastole without major dissipation and was still located at the basal level (Figure 4: f18,f19). For the three remaining subjects, (subjects A, B and C in Table 1) no vortex ring core was present during late diastolic filling. Samples of the Lambda2-based detected peak early and late diastolic formed vortex ring cores are shown in Figure 3 and are depicted together with streamlines visualization of the velocity vector field in Figure 5. A time-sequence of the 3D vortex detection during the diastole is shown in Figure 4 (Additional file 1 and Additional file 2).

### 3D Quantification of LV vortex ring core parameters

The quantified CLR parameters are presented in Table 2. The centers of the 3D vortex ring cores during early and late filling were located at the LV basal level, but the rings during A-filling were significantly closer to the mitral valve compared to the rings during E-filling (E-vortex L=0.19±0.04 versus A-vortex L=0.15±0.05; p=0.0001). The centers of the vortex rings during both E- and A-filling were located in the anterior and anterolateral segments (E-vortex: C=89±23°, A-vortex: C=100±23°; p=NS). A-filling vortex center was located closer to the ventricle’s long-axis during A-filling compared to E-filling (E-vortex: R=0.27±0.07, A-vortex: R=0.20±0.09, p=0.048). Both E- and A- vortex ring cores were similarly orientated relative to the LV long-axis (E-vortex 71.0±9° versus A-vortex 74±4°; p=NS). E-vortex rings were significantly more circular in shape compared to A-vortex rings (E-vortex: CI=0.79±0.09, A-vortex: CI=0.57±0.06; p<0.001).

**Table 2:** **Vortex quantification parameters**

	**C (clockwise) in degrees**	**L**	**R**	**Vortex Orientation in degrees**	**Vortex CI**
**E-vortex ring**	89°±23°	0.19±0.04	0.27±0.07	71° ± 9°	0.79±0.09
A-vortex ring*	100°±23°	0.15±0.05	0.20±0.09	74° ± 4°	0.57±0.06
**Statistical Significant**	No	p=0.001	p=0.048	No	p<0.001

*In 21 subjects an A-filling vortex was observed. Data are presented as mean ± standard deviation.

### Inter and intra-observer variation

Results of inter- and intra-observer analysis for assessment of relative vortex core position and orientation are presented in Tables 3 and 4. Inter-observer analysis revealed intraclass correlation coefficient higher or equal to 0.96 (all p<0.001), with mean relative unsigned differences ranging between 1.5% and 7%, which was not statistically significant different. The coefficient of variation ranged between 1% and 3%. Intra-observer analysis showed intraclass correlation higher or equal to 0.97 (all p<0.001), with a mean relative unsigned difference ranging between 0.5% and 3%, which was not statistically significant different. The coefficient of variation ranged between 1% and 8%.

**Table 3:** **Inter observer analysis for C, relative L, relative R and orientation of vortex ring cores**

	**C**	**L**	**R**	**Vortex Orientation**
**Intraclass correlation (absolute agreement)**	0.940 (p<0.001)	0.976 (p<0.001)	0.964 (p<0.001)	0.985 (p<0.001)
**Mean difference**	0.8	0.002	0.003	0.286
**Confidence interval difference**	−2.4;4.0	−0.003;0.006	−0.006;0.013	−0.239;0.811
**p value difference**	0.62	0.42	0.49	0.28
**Mean relative unsigned difference**	7%	4%	6%	1.5%
**Coefficient of variance**	11%	9%	13%	3%

**Table 4:** **Intra-observer analysis for C, relative L, relative R and orientation of vortex ring cores**

	**C**	**L**	**R**	**Vortex Orientation**
**Intraclass correlation (absolute agreement)**	0.980 (p < 0.000)	0.985 (p < 0.000)	0.988 (p < 0.000)	0.971 (p < 0.000)
**Mean difference**	1.7	0.000	0.001	0.46444
**Confidence interval difference**	−0.1;3.6	−0.003;0.003	−0.004;0.007	−0.279;1.208
**p value difference**	0.07	0.90	0.58	0.21
**Mean relative unsigned difference**	3%	2%	3%	1%
**Coefficient of variance**	7%	7%	7%	3%

### Vortex-Mitral flow association

**Figure 6:**
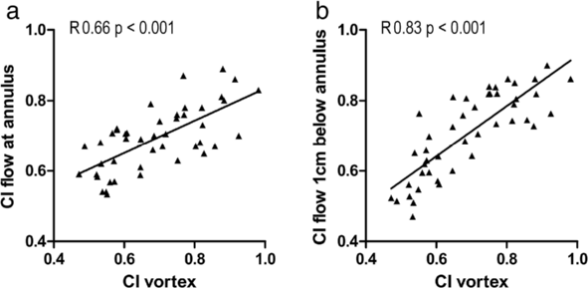
**Correlation between the shape of the formed vortex ring cores (CI vortex) and the shape of the inflow area at the level of both the mitral annulus (a) and the tip of the opened MV leaflets approximated as 1 cm below the annulus (b).**

The Spearman correlation coefficient between the shapes of the vortex ring (CI_vortex_) and the MV inflow jet at the level of the annulus (CI_MV_) was R=0.66 (p<0.001). The correlation coefficient between CI_vortex_ with the shape of inflow jet at the tip of the valve leaflets (CI_MV_tip_) was higher with R=0.83 (p<0.001) (Figure 6).

## Discussion

To our knowledge this is the first work to provide standardized quantitative characterization and comparison of the 3D LV vortex rings during both early and late diastolic filling in normal subjects. Using retrospective-gated 4D Flow CMR and 3D vortex core analysis, using the Lambda2-method, we observed the formation of a separate compact 3D vortex ring *in vivo* during late diastolic filling with different characteristics from the vortex ring formed during early filling. Our experiments quantitatively confirmed the close correlation between the shape of the formed vortex ring and the shape of the inflow area through both the mitral annulus and the tip of the opened MV leaflets.

### LV vortex ring formation and dynamics with emphasis on late diastolic filling

Several studies have demonstrated the presence of rotating flow distal to the MV corresponding to a compact vortex ring during the early diastolic filling. This vortex formation has been related to the normal shape and function of the LV and its alteration has been suggested to be associated with pathologies of the left ventricle [1,4-6,9,11,18,24,25]. In agreement with previous studies, in all subjects a compact 3D vortex ring core was identified distal to the mitral valve during the early filling phase of diastole [6,9,10,18]. In previous studies, vortex analysis within LV flow has been primarily devoted to the early phase of the diastolic filling [1,4-10,15,21,34,35]. Discrepancies exist in literature when defining or evaluating vortex formation during late diastolic filling, where CFD simulation reports vortex ring formation [12-17] and *in vivo* studies report no vortex ring formation but only a single vortex (rotating flow) distal to the anterior MV leaflet [6,9,19-21,36,37] or no vortex at all [18]. Some of the discrepancies among *in vivo* studies can be a result of limitations of the employed flow acquisition and/or analysis approach. 4D Flow CMR has intrinsic advantages over other *in vivo* flow imaging modalities such as Doppler Echocardiography or 2D phase contrast CMR, by allowing acquisition of all three directional velocity components and over the three spatial dimensions. Moreover, 4D Flow CMR provides the feasibility of retrospective flow acquisition therefore allowing acquisition of flow over both early and late diastolic filling phases instead of only the early filling phase as with prospective flow acquisition. Previous studies have successfully employed 4D Flow CMR to visualize and study LV vortex flow [6,19,22,24-27,36,37]. In these studies no explicit analysis of vortex ring formation during late diastolic filling have been performed and relatively low temporal resolution (50–70 ms) were generally used [24,27,36] while higher temporal resolution of 30 ms was used in this study to help capturing flow over the short duration of the late filling (five late filling phases were reconstructed on average).

In the current study, in agreement with CFD findings [12-17], in the majority of subjects (twenty-one subjects, 88%), a compact vortex ring core formed distal to the mitral valve during late diastolic filling. This ring formed at the basal level at the time when the remnant of the dissipating E- vortex ring core was located more apically, indicating that the A-vortex ring is a newly formed vortex as a result of the atrial contraction inflow and not just a continuation of the E-vortex. The A-vortex ring core was asymmetrically shaped in the anterior-posterior direction with a dilated anterior side, making most of the A-vortex flow being located close to the left ventricular outflow tract. This supports the postulation of Kilner et al.[6], about an expected role of the rotating flow beneath the mitral valve during the A-filling in aiding the redirection of the late diastolic inflow from the left atrium towards the left ventricular outflow tract, helping in an optimized ejection of blood. Therefore, with the revealed consistent formation of compact late diastolic vortex ring *in vivo,* extending the analysis of diastolic vortex formation to the late diastolic filling (instead of currently being limited to early filling) might help providing more understanding of the hemodynamics of the coupling between diastole and systole and associated pathologies. This emphasizes the importance of using retrospective cardiac gating when aiming for LV diastolic vortex flow analysis, where late filling phase can be acquired instead of the prospective-gating where late filling phase is generally missing.

The absence of vortex ring formation during late filling in three subjects (Table 1) might be attributed to their age related high heart rate and subsequent limited diastasis duration which might not allow developing the ventricular pressure gradient required for vortex formation [1].

### Quantitative characterization of 3D diastolic vortex rings

Previous studies have successfully employed flow visualization techniques to identify LV vortex flow [6,19-23,26], quantify vortex volume [24] or evaluate early filling vortex formation [35]. However, to our knowledge, there have been no *in vivo* studies providing quantitative 3D characterization of the location and the shape of vortex flow during both early and late diastolic filling phases.

Defining the true boundary of a vortex is challenging task, especially in 3D space, as it is highly dependent on the identifier. Most *in vivo* studies identify a vortex based on visual assessment of the visualized flow [9,11,20,21] which is generally an observer dependent definition. Instead, vortex cores are generally regarded as a robust and well localized approximation of a vortex [2,3,28,38] and can provide more objective physical definition of a vortex. Different methods can be used for vortex core identification [13,28-30], however, the Lambda2-method is considered the most accepted 3D vortex identification technique [1]. Vortex core analysis has been used before to detect vortices inside the heart but mainly for visualization purposes [10,13,27,29,32,39]. In this work, we employ the 3D vortex cores identified using the Lambda2-method to derive quantitative parameters to characterize normal vortex ring formation during both peak early and peak late filling. In our experiments, following [32], Lambda2 isovalue threshold (${\mathrm{T}}_{\lambda_2}$) in the range of [1,6] μ (i.e. **K** = [1, 6], with μ as the λ_2_ average of voxels with λ_2_ < 0) allowed identification of a separate compact vortex ring core (when detected) in all subjects. The strong inter- and intra-observer agreements (Tables 3 and 4) indicate the robustness of the method with respect to Lambda2 threshold selection.

The vortex ring core is significantly closer to the mitral valve annulus (longitudinal position) at the late filling peak compared to early filling, which can be attributed to the lower velocity and shorter length of inflow jet during late filling compared to early filling. The relatively closer position of the vortex ring core to the LV long-axis (radial position) at the late filling, can be explained using the confirmed correlation between shapes of the vortex ring core and the mitral valve opening, where a restricted opening of the mitral valve during late filling results in a vortex core center closer to the long-axis compared to full valvular opening at the early filling. Since vortex ring originates from the inlet jet at the distal tip of the mitral valve (MV) leaflets [1,10,13], vortex ring forms parallel to the inclined MV orifice [40]. Therefore, in normal subjects, similarly oriented MV orifice of early and late filling (relative to the ventricle’s long-axis) results in similarly oriented vortex rings (i.e. similar vortex orientation planes). Consequently, circumferential location (C), which is calculated using the vortex orientation plane, is similar as well between vortex rings of both early and late filling. The strong correlation between the vortex ring shape with the shape of the inflow area at the tip of the opened MV leaflets confirms the relationship between the mitral valvular opening and shape of formed vortex ring as reported earlier by CFD studies [4,13]. To the best of our knowledge, this is the first *in vivo* study to quantitatively confirm this correlation.

The relatively small variation between normal subjects in derived parameters (Table 2) indicates good consistency of results. Therefore, the method defines normal quantitative ranges for diastolic vortex rings and might in future help evaluating whether changes in valve morphology or ventricular dilatation alters the location and shape of the formed vortices.

### Clinical implications

The suggested LV-normalized vortex parameters might help to provide more insights about the normal vortex formation and provide normative parameters to compare the 3D vortex flow between controls and patients. This could help to understand the hemodynamics of patients with impaired LV relaxation and restrictive filling, where the E/A ratio is abnormal. In addition, the close correlation found between vortex formation and the flow at the tip of the opened mitral valve leaflets suggests that patients with impaired leaflet function, as can be seen in patients with left ventricular dysfunction [41] after mitral valve repair and with mitral valve stenosis, could develop aberrant vortex rings, which possibly reduces efficiency of intra-cardiac flow. Therefore, further study is warranted to investigate the effect of mitral valve surgery on vortex formation during LV filling.

### Study limitations

Limitations of this study include a relatively small number of healthy subjects and lack of comparison with patients. However, an objective detection of possible anomalies in the vortex flow of patients should be preceded by finding reliable quantitative measures defining the reference normal vortex flow. The current study was performed in a relatively young population (age range 9 – 44 years). Global diastolic function parameters, as the E/A ratio remain relatively stable during the second, third and fourth decade of life [42], which explains why we did not observe age related differences. As diastolic function is known to decrease later in life [43] future studies are required to compute normal values in an elderly population. Limitations of 4D Flow CMR include the relatively long scan times (typically between 8–10 minutes with heart rate 60-80bpm), and the need of averaging the data over several cardiac cycles. This time-averaging would potentially result in smoothing the low scale flow structures and does not, generally, account for flow variations due to heart rate variations. In this study, a relatively low spatial CMR resolution of 2.3×2.3×3-4 mm^3^ was used. However, it was our aim to evaluate large scale vortex ring cores which are expected to have volumes significantly larger than the MR voxel size. In three volunteers, a higher resolution of 2.3×2.3×3 mm^3^ was used, which did not result in significant different findings from the other subjects. Further methodological and quantitative analysis on the effect of acquisition resolution may be helpful but was beyond the scope of this work. Identified Lambda2-based vortex cores could not be used for volumetric measurements (e.g. vortex volume or size) as applying different Lambda2 isovalue thresholds can result in different volumes for the same vortex core. Therefore, the vortex parameters derived in this study were chosen as not to be dependent on vortex volume. 4D Flow data was acquired using free breathing scans and no motion compensation was applied. Nevertheless, no motion artifacts were visually observed in the velocity data, and since all subjects underwent the same scan protocol, potential inter- and intra-subject effects on the measurements might be assumed to be similar among all subjects.

## Conclusion

In summary, this is the first *in vivo* study using 4DFlow CMR to confirm previous CFD findings of vortex ring formation during late filling and to provide standardized parameters that allow quantitative characterization of vortex flow during both early and late left ventricular filling. The derived quantitative parameters provided consistent measurements within the studied population and strong correlation was found between the shape of the formed vortices and the shape of the inflow area at the level of both the mitral annulus and the tip of the opened MV leaflets. This study provides reference parameters defining normal vortex flow, which may allow objective quantitative evaluation of vortex flow in patients with cardiac disease.

## Additional files

Additional file 1:
**Three dimensional Left ventricular diastolic vortex formation and development over the entire diastole in a sample healthy subject.** Movie showing 3D left ventricular vortex formation and development, as detected by the Lambda2 method and visualized as isosurfaces in red color, over all 19 acquired diastolic phases in a sample normal subject, with E-filling onset (x), peak (y) and end (z), and A-filling onset (u), peak (v) and end (w). Diastasis is the duration between z and u. Every dot in the cardiac curve corresponds to a time point of the cardiac cycle in which a 4D Flow volume was acquired. To avoid cluttered view, only large scale vortex cores of 1 cm^3^ or larger are visualized. Lambda2 isovalue threshold $\left({\mathrm{T}}_{\lambda_2}\right)=3\upmu$ was used to define the isosurfaces of vortex ring cores (with μ as the λ_2_ average of voxels with λ_2_ < 0). Additional file 2:
**3D Vortex cores superimposed on streamline visualization over the entire diastole in a sample healthy subject.** Movie showing vortex cores of Additional file 1 superimposed on streamline visualization of a segmented LV cross-sectional view showing good overlap between the 3D Lambda2-detected vortex cores and the cores of corresponding 2D streamlines’ counter-rotating vortices during both early and late diastolic filling.
